# Adherence to the EAT-Lancet diet in midlife and development in weight or waist circumference after five years in a Danish cohort

**DOI:** 10.1016/j.dialog.2023.100151

**Published:** 2023-08-19

**Authors:** Fie Langmann, Daniel B. Ibsen, Anne Tjønneland, Anja Olsen, Kim Overvad, Christina C. Dahm

**Affiliations:** a Department of Public Health, Aarhus University, Bartholins Allé 2, 8000 Aarhus, Denmark; b Steno Diabetes Center Aarhus, Aarhus University Hospital, Palle-Juul Jensens Blvd. 11, Entrance A, 8200 Aarhus, Denmark; c Department of Nutrition, Exercise and Sports, University of Copenhagen, Nørre Allé 51, 2100 Copenhagen, Denmark.; d Danish Cancer Society, Strandboulevarden 49, 2100, Copenhagen, Denmark; e Department of Public Health, University of Copenhagen, Øster Farimagsgade 5, 1353 Copenhagen, Denmark

**Keywords:** Cohort study, Epidemiology, Sustainable diet, Sustainable dietary patterns, Weight change, Waist circumference

## Abstract

**Purpose:**

The EAT-Lancet reference diet has been proposed as a healthy dietary pattern to reduce food-related climate impacts, but little is known regarding associations with bodyweight development. This study investigated adherence to the EAT-Lancet diet in midlife and development in weight and waist circumference (WC) after five years.

**Design:**

The Danish Diet, Cancer and Health cohort recruited participants in 1993–1997. At baseline, data on diet, lifestyle, and anthropometry were collected. Participants self-reported weight and WC five years later. In total, 44,194 participants were included in analyses of weight (43,678 for WC). Baseline adherence to the EAT-Lancet diet was scored 0–14 points. Multiple linear regression was used to estimate associations between the EAT-Lancet diet and development in weight and WC after five years. Poisson regression was used to estimate risk ratios (RR) of obesity (≥30 kg/m^2^) or elevated WC.

**Results:**

Adherence to the EAT-Lancet diet was not associated with follow-up weight, adjusting for baseline weight and confounders (11–14 vs 0–7 points β: -0.08, 95% CI: -0.27, 0.11 kg), but was associated with lower follow-up WC adjusting for baseline WC and confounders (β: -0.38, 95% CI: -0.69, -0.07 cm), and was associated with lower risk of obesity and elevated WC (RR 0.89, 95% CI: 0.82, 0.98, and 0.95, 95% CI: 0.93, 0.96, respectively).

**Conclusion:**

Adherence to the EAT-Lancet diet in midlife was associated with lower WC but not weight after five years follow up, taking baseline into account. Our findings suggest that greater adherence to the EAT-Lancet diet does not contribute to development of obesity.

## 1 Introduction

Over the past decades there has been a global surge in the prevalence of obesity, which is associated with a higher risk of several non-communicable diseases such as cardiovascular diseases, cancers, and type 2 diabetes [1,2]. Dietary patterns are considered modifiable risk factors in prevention and treatment of obesity, and with the global syndemic of obesity, malnutrition and climate change, there is great attention on the urgency for a shift in our current dietary patterns towards healthy and sustainable diets [3].

The Lancet Commission on Planetary Health proposed the EAT-Lancet reference diet in 2019 as a sustainable dietary pattern that is both healthy and within the boundaries of planetary resources [4]. The diet emphasizes higher intakes of fruits, vegetables, plant-based protein and unsaturated fats, and less red meats, than standard Western dietary patterns [4,5]. This diet could be a potential candidate for tackling the global syndemic, if recommended and implemented by political authorities and valued as urgent and accepted by the public [3].

The EAT-Lancet diet resembles a vegetarian diet, which in previous research has shown to be associated with lower risk of obesity and obesity-related diseases compared to non-vegetarian diets [6,7]. High consumption of meats, particularly red and processed, compared with consumption of other foods, has also been associated with higher risk of weight gain and obesity [8]. Nonetheless, very few studies have evaluated the EAT-Lancet diet in relation to long-term weight management, and none in a Danish setting, although the new Danish dietary recommendations are founded on the EAT-Lancet diet [4,9,10].

Therefore, this study aimed to investigate whether high adherence to the EAT-Lancet diet compared to low adherence was associated with 5-year follow-up weight and waist circumference (WC) in middle-aged Danish men and women. We further aimed to use several ways of analysing weight and WC change scores, each with different interpretations, to assess different assumptions about the associations.

## 2 Subjects and methods

### 2.1 Study population

Eligible individuals for the Danish Diet, Cancer and Health Cohort (DCH) had to be born in Denmark, live in Copenhagen or Aarhus County, be 50–64 years old at inclusion, and have no previous diagnosis of cancer in the Danish Cancer Registry. Of the 160,725 eligible invitees during the recruitment period in 1993–1997, 57,053 participated in the study [11]. Participants completed questionnaires on diet and lifestyle and visited one of two study centres for anthropometric and other biological measurements. Questionnaires were optically scanned at the study centre to check for errors and missing information. Afterwards, a lab technician clarified all unclear information with participants. The Danish Data Protection Agency and the local ethical committees of Copenhagen and Frederiksberg Municipalities (in Danish: "Den Videnskabsetiske komite for Københavns og Frederiksberg Kommuner") approved the study with approval no.: (KF) 01-345/93. All participants gave written informed consent [11].

Participants who had cancer before baseline, erroneously invited due to processing delay at the Cancer Registry, had missing or incomplete data on diet, or had missing data on covariates were excluded.

### 2.2 Assessment of the EAT-Lancet diet score

Prior to the visit to one of the study centres, dietary data were collected using a 192-item self-administered food frequency questionnaire (FFQ) [11,12]. Participants reported their average intake of different food and beverage items over the past 12 months within 12 possible categories ranging from never to eight times or more per day. Intake was estimated using national food composition database and a specifically designed software program, FoodCalc version 1.3 [11,13]. The FFQ was validated against two weighed diet records of seven consecutive days filled in by a random sample of 40–64-year-old men and women from Copenhagen. Approximately 70% of subjects were classified in the same quintile of nutrient intake distribution in the FFQ compared with the diet records [12].

This study used an EAT-Lancet diet score previously constructed by Knuppel et al. [14]. Adherence to the EAT-Lancet diet was scored based on estimated average daily intake of 14 dietary components (Supplemental Table 1). Each component contributed 0 (non-adherence) or 1 point (adherence), resulting in a total score ranging 0–14 points. Adherence was categorized in quintiles with cut-points at 0–7 points (reference group), 8 points, 9 points, 10 points, and 11–14 points.

### 2.3 Assessment of weight and WC

Trained personnel measured participants’ weight, height, and WC at baseline. Weight was measured to the nearest 0.1 kg with Soehnle digital scales. WC was measured to the nearest 0.5 cm at the natural waist or midway between lowest rib and iliac crest with a non-stretchable measuring tape. Height was measures to the closest 0.5 cm standing without shoes [11]. Participants still living in Denmark approximately five years after the first data collection received a follow-up questionnaire collecting information on self-reported weight and WC. Participants were asked to weigh themselves in light underwear. They also received a measuring tape to measure their WC, and to ease the measurement, they were instructed to measure at the belly button [11]. Self-reported waist measurement was validated as a useful proxy for professionally measured WC within the cohort. Spearman correlation coefficients between the professionally measured WC at the natural waist and the self-reported WC measure at the belly button were 0.88 in men and 0.86 in women [15].

### 2.4 Assessment of covariates

Data on lifestyle components were collected through a self-administered lifestyle questionnaire at the study centre during the baseline assessment and included questions on smoking history, educational level, physical activity, and previous history of hypertension, diabetes, hypercholesterolemia, stroke, and acute myocardial infarct (AMI) [11]. Information on biological sex was obtained through the Danish Civil Registration System by using the unique sex-specific compulsory 10-digit-personal identification number assigned to each participant at birth or in 1968, when the registry was created [16]. Information on alcohol intake was assessed through the FFQ [17]. Potential confounders were chosen *a priori* based on a review of the literature and Directed Acyclic Graphs [18] presented in Supplemental Fig. 1.

### 2.5 Statistics

Standard summary statistics were used to describe characteristics across categories of adherence to the EAT-Lancet diet.

**Fig. 1:**
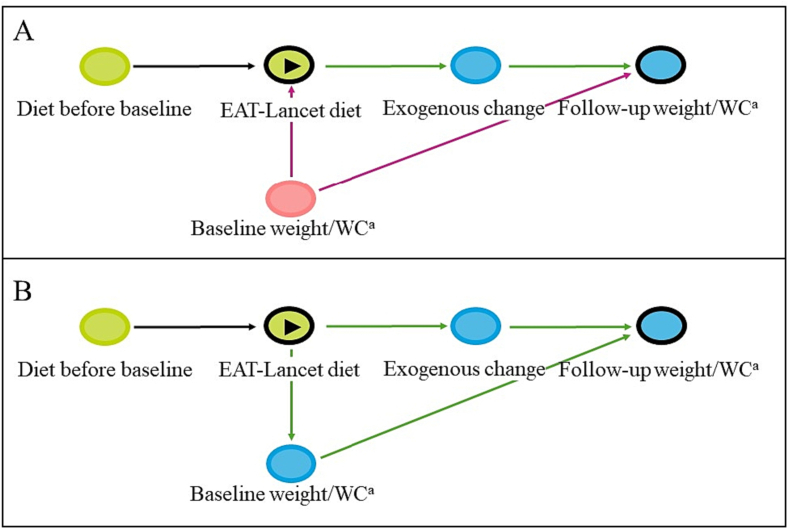
Directed Acyclic Graph (DAG) of the association between diet before study baseline and baseline measures of weight and waist circumference and EAT-Lancet diet score at baseline. This DAG is constructed in DAGitty [20]. ^a^WC, Waist circumference,  exposure,  outcome,  ancestor of exposure,  ancestor of outcome  ancestor of exposure *and* outcome,  causal path,  biasing path. Panel A represents baseline weight and WC as confounders for the causal association between EAT-Lancet diet and follow-up weight and WC. The direct causal association between EAT-Lancet diet and follow-up weight and WC is the same as the direct causal association between the exogenous change and follow-up weight and WC. Panel B represents baseline weight and WC as mediators of the causal association between EAT-Lancet diet and follow-up weight and WC. The total causal association represents the direct association between the exogenous change and follow-up weight and WC, and the indirect association mediated through baseline weight and WC.

Multivariable-adjusted linear regression models were used to estimate the association between adherence to the EAT-Lancet diet and follow-up weight and WC after five years. The primary outcomes for this study were weight and WC at follow-up adjusted for the respective baseline measures of the two (model 1a). This adjustment would estimate the direct effect of different levels of EAT-Lancet adherence during the follow-up period, under the assumption that baseline weight or WC were confounders [19]. Conditioning on baseline weight and WC could eliminate their potential confounding of the association of interest and restrict the estimated association to only include the direct effect of baseline diet on follow-up weight and WC, mimicking a randomized design [19]. This follows an assumption that participants have reached a steady state in their diet before entry to the study, due to their age and life conditions. Under this assumption baseline weight and WC and baseline diet would be affected by diet prior to baseline, and therefore baseline weight and WC could equally act as confounders (Fig. 1, panel A) or mediators (Fig. 1, panel B) of the association between the diet at baseline and the follow-up measures of weight and WC.

Our primary assumption was that baseline weight and WC were confounders (Fig. 1, panel A). Model 1b was further adjusted for potential confounders measured at baseline: sex (male; female), age (years), education (vocational; 1–2 years, 3–4 years, >4 years), physical activity ≥30 min/day (yes; no), smoking status (never, previous, current), alcohol intake (g/day), and history of hypertension (yes; no; don’t know), hypercholesterolemia (yes; no; don’t know), diabetes (yes; no; don’t know), stroke (yes; no), and AMI (yes; no) before baseline. Model 2 was further adjusted for total energy intake (kcal/day). Results are presented as estimates (β) with corresponding 95% confidence intervals (CI).

Since it could be argued that baseline weight and WC are mediators of the relation between adherence to the EAT-Lancet diet at baseline and development in weight and WC after five years (See Fig. 1), analyses were also conducted without adjustment for baseline weight and WC. The estimated associations from these analyses can be interpreted as the total effect of adherence to the EAT-Lancet diet at baseline on weight and WC at follow-up (Fig. 1, panel B) [19]. Thus, part of the estimated effect will be due to the direct effect of baseline diet on follow-up weight and WC and part of the effect will be due to the indirect effect of baseline diet on follow-up weight and WC, mediated through baseline weight and WC, under the assumption of dietary steady state in participants.

To assess linearity, the EAT-Lancet score was modelled as a restricted cubic spline. Potential effect modification by baseline weight or WC, age, and sex was investigated in stratified analyses. For continuous variables, except WC, the median was used as the cut-point for the strata. For WC the sex-specific cut-offs for low risk of cardiovascular disease of 80 cm for women and 94 cm for men were used as cut-point for the strata [21].

Multivariable-adjusted modified Poisson regression models were used to estimate the risk ratio (RR) and 95% CI of obesity (BMI ≥ 30 kg/m^2^) at follow-up across EAT-Lancet categories, among individuals with BMI < 30 kg/m^2^ at baseline, and for the risk of elevated WC (female ≥80 cm; male ≥94 cm) at follow-up across EAT-Lancet categories, among individuals without elevated WC at baseline. The cut-off for obesity set by the WHO (BMI ≥ 30 kg/m^2^) was used in this study, since only obesity, and not overweight, has been associated with higher mortality risk [1,2]. Sex-specific cut-offs for WC for low risk of cardiovascular disease of 80 cm for women and 94 cm for men were used, as recommended by the WHO [21]. Robust error variance procedure was applied in the analyses to help minimize overestimation of the risk, which is often observed when applying logistic regression models in cohort studies and interpreting the odds ratio as risk ratio [22]. The analyses were adjusted as in model 1b.

In sensitivity analyses, potential outliers in follow-up weight and WC were removed, as they were self-assessed by participants. Outliers were defined as individuals with WC <30 cm at follow-up, change in WC from baseline to follow-up of ≥50 cm, or changes in weight from baseline to follow-up of ≥40 kg. Because some participants were lost to follow-up between baseline and follow-up, characteristics at baseline of participants who participated in both assessments and those who only participated at baseline but could have participated at follow-up were compared. Inverse probability weights were used to adjust for non-participation at follow-up. The following covariates measured at baseline were used to predict non-participation at follow-up: WC, weight, EAT-Lancet score, sex, age, educational level, physical activity, smoking status, alcohol intake, and history of hypercholesterolemia, diabetes, hypertension, AMI, or stroke. Development of certain diseases between baseline and follow-up could influence follow-up weight and WC. Therefore, sensitivity analyses excluding all participants who developed diabetes, AMI, stroke, and colorectal cancer during follow-up were conducted. Adherence to the EAT-Lancet diet was crudely scored with only two options for adherence (yes or no), and sensitivity analyses were therefore conducted with a more gradual scoring based on Stubbendorff’s EAT-Lancet index [23] to investigate the robustness of the estimated associations between EAT-Lancet diet and follow-up weight and WC. The adjustment levels for all sensitivity analyses were the same as in model 1b.

Since measures of changes in weight and WC are often used as outcomes in other studies, changes in weight and WC as the outcome with and without adjustment for baseline weight or WC were also investigated. The adjustment level for the change analyses were the same as in model 1b.

The significance level was set at ≤5% and all analyses were conducted using StataIC version 17.0 (StataCorp LLC, College Station, Texas, USA) [24].

## 3 Results

### 3.1 Study population

**Fig. 2:**
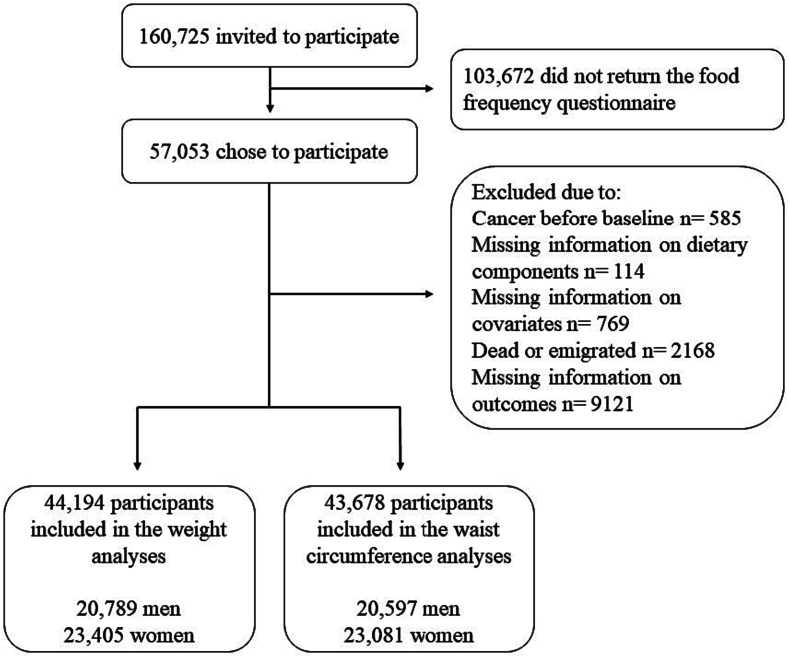
Flow diagram of participants from the Danish Diet, Cancer and Health cohort eligible for the statistical analyses.

The study included 44,296 of the 57,053 eligible participants in DCH after exclusion of individuals with missing information on diet, weight, height, WC, or covariates (Fig. 2).

At baseline, participants with higher adherence to the EAT-Lancet diet were more likely to be female, to not smoke, have a longer education, consume less alcohol, and were more likely to have a history of hypertension, diabetes, and hypercholesterolemia. Weight, BMI, and WC at baseline was lower among participants with a high EAT-Lancet score compared to those with a low score. The average WC was above the recommended levels for both sexes independent of EAT-Lancet score (Table 1).

**Table 1:** Baseline characteristics of participants across EAT-Lancet score categories.

EAT-Lancet score	Total population N = 44,296	0–7 points n = 4803	8 points n = 9652	9 points n = 13,221	10 points n = 10,342	11–14 points n = 6278
Female, n (%)	23,463 (53.0)	1491 (31.0)	3992 (41.4)	6809 (51.5)	6514 (63.0)	4657 (74.2)
Age (years)	56 (51–63)	56 (51–63)	56 (51–63)	56 (51–63)	55 (51–62)	55 (51–62)
Educational level, n (%)						
Vocational	5909 (13.3)	804 (16.7)	1453 (15.1)	1835 (13.9)	1204 (11.6)	613 (9.8)
Short (1–2 years)	10,071 (22.7)	938 (19.5)	2041 (21.2)	2998 (22.7)	2482 (24.0)	1612 (25.7)
Medium (3–4 years)	18,193 (41.1)	2029 (42.2)	4016 (41.6)	5334 (40.3)	4268 (41.3)	2546 (40.6)
Long (>4 years)	10,123 (22.9)	1032 (21.5)	2142 (22.2)	3054 (23.1)	2388 (23.1)	1507 (24.0)
Current smokers, n (%)	14,585 (32.9)	2072 (43.1)	3642 (37.7)	4452 (33.7)	2962 (28.6)	1457 (23.2)
Weight (kg)	74.3 (58.5–93.4)	78.2 (60.8–96.4)	76.3 (59.9–94.7)	74.5 (58.7–93.8)	72.4 (57.7–91.7)	70.1 (57.0–90.1)
BMI (kg/m^2^)	25.4 (21.4–30.9)	25.9 (21.7–31.0)	25.7 (21.7–30.9)	25.5 (21.5–31.0)	25.3 (21.3–30.8)	24.9 (21.2–30.7)
WC^a^ (cm)						
Women	80.0 (69.0–97.0)	81.0 (69.5–99.0)	80.0 (69.5–90.0)	80.0 (70.0–97.0)	80.0 (69.0–96.0)	79.0 (69.0–95.0)
Men	95.0 (84.0–108.0)	95.0 (84.0–109.0)	95.0 (84.0–108.0)	95.0 (84.0–108.0)	95.0 (84.0–107.0)	94.0 (83.0–107.0)
Physical activity ≥ 30 min/day, n (%)	13,893 (40.4)	1847 (38.5)	3685 (38.2)	5173 (39.1)	4301 (41.6)	2887 (46.0)
Alcohol intake (g/day)	13.0 (1.7–45.7)	14.5 (1.8–55.3)	13.6 (1.8–50.6)	13.5 (1.8–47.1)	12.6 (1.7–43.4)	11.6 (1.5–39.2)
Energy intake (MJ/day)	8.90 (6.17–12.47)	10.82 (8.00–14.84)	9.74 (7.07–13.25)	8.9 (6.28–12.21)	8.23 (5.76–11.29)	7.59 (5.42–10.13)
History of						
Hypertension, n (%)	6949 (15.7)	639 (13.3)	1461 (15.1)	2139 (16.2)	1649 (15.9)	1061 (16.9)
Hypercholesterolemia, n (%)	3234 (7.3)	282 (5.9)	667 (6.9)	976 (7.4)	825 (8.0)	484 (7.7)
Diabetes, n (%)	808 (1.8)	51 (1.1)	142 (1.5)	228 (1.7)	208 (2.0)	179 (2.9)
Stroke, n (%)	366 (0.8)	40 (0.8)	72 (0.8)	124 (0.9)	83 (0.4)	47 (0.8)
AMI^b^, n (%)	560 (1.3)	74 (1.5)	136 (1.4)	177 (1.3)	112 (1.1)	61 (1.0)

Distributions are expressed as medians and 10–90% percentiles unless otherwise specified.

a WC, Waist circumference.

b AMI, acute myocardial infarction.

### 3.2 Association between EAT-Lancet score and follow-up weight and WC

The primary analysis of follow-up weight adjusted for baseline weight confounders (Table 2, model 1b) showed no difference in weight at follow-up among participants with the highest EAT-Lancet score (11–14 points) compared with the lowest (0–7 points) (β: -0.08, 95% CI: -0.27, 0.11 kg). This was also the case after further adjustment for energy intake. The primary analysis of follow-up WC conditioned on baseline WC and confounders (Table 2, model 1b), showed a lower WC at follow-up among participants with the highest EAT-Lancet score compared to those with the lowest (β: -0.38, 95% CI: -0.69, -0.07 cm). WC was lower after further adjustment for energy.

**Table 2:** Association between EAT-Lancet score and weight or waist circumference (WC) at follow-up adjusted for baseline weight or WC.

	β (95% CI)				
EAT-Lancet score	0–7	8	9	10	11–14
Mean weight difference at follow-up (kg)^a^	-0.07 (-0.21, 0.06)	-0.22 (-0.32, -0.12)	-0.20 (-0.29, -0.11)	-0.38 (-0.47, -0.29)	-0.27 (-0.39, -0.15)
Model 1a^b^	Reference	-0.25 (-0.43, -0.08)	-0.30 (-0.47, -0.14)	-0.58 (-0.75, -0.41)	-0.57 (-0.76, -0.38)
Model 1b^c^	Reference	-0.12 (-0.29, 0.05)	-0.05 (-0.22, 0.11)	-0.19 (-0.37, -0.02)	-0.08 (-0.27, 0.11)
Model 2^d^	Reference	-0.13 (-0.30, 0.05)	-0.07 (-0.24 0.10)	-0.22 (-0.40, -0.04)	-0.11 (-0.32, 0.10)
Mean WC difference at follow-up (cm)^e^	4.3 (4.0, 4.5)	4.6 (4.5, 4.8)	5.1 (4.9, 5.2)	5.5 (5.4, 5.7)	5.9 (5.7, 6.1)
Model 1a^b^	Reference	-0.06 (-0.33, 0.22)	0.07 (-0.20, 0.33)	0.05 (-0.23, 0.32)	-0.07 (-0.37, 0.24)
Model 1b^c^	Reference	-0.14 (-0.41, 0.14)	-0.10 (-0.36, 0.16)	-0.19 (-0.47, 0.08)	-0.38 (-0.69, -0.07)
Model 2^d^	Reference	-0.17 (-0.45, 0.10)	-0.16 (-0.43, 0.11)	-0.28 (-0.57, 0.02)	-0.48 (-0.81, -0.16)

a N = 44,194.

b Multi-variable linear regression analyses adjusted for baseline measures of weight or WC respectively.

c Further adjusted for sex (male, female), age at inclusion (years), physical activity (≥30 min/day, <30 min/day of moderate-to-vigorous physical activity), education (vocational, short 1–2 years, medium 3–4 years, high >4 years), smoking status (never, former, current <15 g tobacco/day, current 15–25 g tobacco/day, current >25 g tobacco/day), alcohol intake (g/day, restricted cubic splines with 4 knots), and previous history of hypertension (yes, no, don’t know), hypercholesterolemia (yes, no, don’t know), diabetes (yes, no, don’t know), stroke (yes, no), and acute myocardial infarction (yes, no) before baseline.

d Further adjusted for energy intake (kJ/day, continuous).

e N = 43,678.

Assuming that baseline weight and WC were mediators of the association between EAT-Lancet adherence and follow-up weight and WC (Fig. 1, panel B), and thus should not be controlled for, the non-baseline adjusted analysis (Table 3) showed a lower follow-up weight in the highest EAT-Lancet category compared to the lowest (β: -0.92, 95% CI: -1.38; -0.45 kg). Follow-up WC without adjustment for baseline WC was also lower in the highest EAT-Lancet category compared to the lowest (β: -1.54, 95% CI: -1.98; -1.10 cm, Table 3).

**Table 3:** Association between EAT-Lancet score and different models assessing difference in weight and waist circumference (WC).

	β (95% CI)				
EAT-Lancet score	0–7	8	9	10	11–14
Follow-up weight^a^					
with baseline adjustment	Reference	-0.12 (-0.29, 0.05)	-0.05 (-0.22, 0.11)	-0.19 (-0.37, -0.02)	-0.08 (-0.27, 0.11)
w/o^b^ baseline adjustment	Reference	-0.52 (-0.93, -0.11)	-0.48 (-0.88, -0.09)	-0.73 (-1.14, -0.31)	-0.92 (-1.38, -0.45)
Follow-up WC^c^					
with baseline adjustment	Reference	-0.14 (-0.41, 0.14)	-0.10 (-0.36, 0.16)	-0.19 (-0.47, 0.08)	-0.38 (-0.69, -0.07)
w/o baseline adjustment	Reference	-0.48 (-0.87, -0.09)	-0.50 (-0.88, -0.12)	-0.81 (-1.21, -0.42)	-1.54 (-1.98, -1.10)

Multivariable linear regression analyses adjusted for sex (male, female), age at inclusion (years), physical activity (≥30 min/day, <30 min/day of moderate-to-vigorous physical activity), education (vocational, short 1–2 years, medium 3–4 years, high >4 years), smoking status (never, former, current <15 g tobacco/day, current 15–25 g tobacco/day, current >25 g tobacco/day), alcohol intake (g/day, restricted cubic splines with 4 knots), and previous history of hypertension (yes, no, don’t know), hypercholesterolemia (yes, no, don’t know), diabetes (yes, no, don’t know), stroke (yes, no), and acute myocardial infarction (yes, no) before baseline.

a Total N = 44,194 included in weight analyses.

b w/o, without.

c Total N = 43,678 included in WC analyses.

Plots of EAT-Lancet score modelled as restricted cubic splines did not dispute linearity in the association between adherence to the EAT-Lancet diet and development in weight or WC (Supplemental Figs 2 and 3). Removal of outliers did not alter the patterns of associations (Supplemental Table 2).

When stratifying by age, sex, baseline weight, and baseline BMI, the associations between EAT-Lancet diet score and weight at follow-up only resulted in different directions of association in the BMI-stratified analyses, while in the other analyses, all strata had similar directions of associations (Supplemental Table 3). Among participants with a baseline BMI below 25 kg/m^2^, highest compared to lowest EAT-Lancet score was associated with a lower follow-up weight (β: -0.28; 95% CI: -0.51; -0.04 kg). For participants with a baseline BMI above 25 kg/m^2^, there was no difference in follow-up weight between individuals with lowest and highest EAT-Lancet adherence (β: 0.06, 95% CI: -0.25; 0.37 kg).

There were similar trends when analyzing follow-up WC across strata of age, sex, baseline WC, and baseline BMI. Only the BMI- and WC-stratified analyses resulted in different directions of association (Supplemental Table 4). Among participants with a baseline BMI below the median, highest compared to lowest EAT-Lancet score was associated with a lower follow-up WC (β: -0.57, 95% CI: -0.97; -0.16 cm). For participants with a baseline BMI above the median, there was no difference in WC between individuals with lowest and highest EAT-Lancet adherence (β: -0.24, 95% CI: -0.70; 0.23 cm). In the baseline WC-stratified analyses highest compared to lowest EAT-Lancet adherence was negatively associated with follow-up WC among participants with baseline WC below the WHO-specified cut-offs (β: -0.52, 95% CI: -0.93; -0.11 cm). No differences in WC between individuals with lowest and highest EAT-Lancet adherence was observed in the strata of those with baseline WC above the cut-offs (β: -0.24, 95%CI: -0.71; 0.24 cm).

### 3.3 Adherence to the EAT-Lancet diet and risk of obesity or elevated WC

For individuals with BMI < 30 kg/m^2^ at baseline (N = 38,278, n developed obesity during follow-up = 1475), the estimated RR for obesity at follow-up for those with highest EAT-Lancet adherence was significantly lower compared to those with lowest EAT-Lancet adherence (RR: 0.89, 95% CI: 0.82, 0.98, Table 4). We found a lower risk of elevated WC at follow-up among those with a WC below the recommended levels at baseline (N = 20,781, n developed elevated WC = 5288, RR: 0.95, 95% CI: 0.93, 0.96 comparing those with the greatest to lowest EAT-Lancet diet score; Table 4).

**Table 4:** Association between EAT-Lancet adherence and risk of obesity^a^ and elevated waist circumference (WC)^b^ at follow-up.

EAT-Lancet score	0–7	8	9	10	11–14
BMI < 30 kg/m^2^ at baseline, n^c^	4097	8317	11,362	9016	5486
Obese^a^ at follow-up, n (%)	175 (4.3)	339 (4.1)	427 (3.8)	337 (3.7)	197 (3.6)
RR for obesity (95%CI)	Reference	0.94 (0.87, 1.02)	0.96 (0.89, 1.03)	0.90 (0.83, 0.98)	0.89 (0.82, 0.98)
WC < cut-offs at baseline, n^d^	1334	3572	5966	5755	4154
Elevated WC^b^ at follow-up, n (%)	401 (30.0)	1000 (28.0)	1547 (25.9)	1414 (24.6)	926 (22.3)
RR for elevated WC (95% CI)	Reference	0.99 (0.98, 1.00)	1.00 (0.99,1.01)	0.98 (0.97, 1.00)	0.95 (0.93, 0.96)

Modified Poisson regression approach [22] adjusted for sex (male, female), age at inclusion (years), physical activity (≥30 min/day, <30 min/day of moderate-to-vigorous physical activity), education (vocational, short 1–2 years, medium 3–4 years, high >4 years), smoking status (never, former, current <15 g tobacco/day, current 15–25 g tobacco/day, current >25 g tobacco/day), alcohol intake (g/day, restricted cubic splines with 4 knots), and previous history of hypertension (yes, no, don’t know), hypercholesterolemia (yes, no, don’t know), diabetes (yes, no, don’t know), stroke (yes, no), and acute myocardial infarction (yes, no) before baseline.

a Individuals with BMI < 30 kg/m^2^ at baseline who have BMI ≥30 kg/m^2^ at follow-up. Percentages express the proportion of individuals who developed obesity at follow-up within each category of EAT-Lancet scores.

b Individuals with waist circumference (WC) below WHO’s sex specific cut-offs of 80 cm for women or 94 cm for men at baseline, who have a WC above the sex specific cut-offs at follow-up [21]. Percentages express the proportion of individuals who developed elevated WC at follow-up within each category of EAT-Lancet scores.

c Total sample N = 38,278.

d Total sample N = 20,781.

### 3.4 Sensitivity analyses

The non-participation analysis (Supplemental Table 5) showed more participants with incomplete follow-up data in the lowest EAT-Lancet category compared to the highest. The inverse probability weighted analyses of the association between EAT-Lancet adherence and weight and WC at follow-up showed similar estimates as the main analyses (Supplemental Table 6).

Exclusion of participants who developed diabetes, AMI, stroke, or colorectal cancer during follow-up (n = 2207) resulted in associations of similar magnitudes as in the main analyses (Supplemental Table 7). When adjusting for baseline weight and confounders (model 1b), those with the highest EAT-Lancet score compared to the lowest had a 0.12 kg lower follow-up weight, albeit with CIs including the null (95% CI: -0.31; 0.08 kg). When further adjusting for energy intake, the association was -0.14 kg (95% CI: -0.34; 0.07 kg). When adjusting for baseline WC and confounders (model 1b), those with the highest compared to the lowest EAT-Lancet score had a lower follow-up WC (β: -0.42, 95% CI: -0.73; -0.10 cm). When further adjusting for energy intake, the association was of slightly greater magnitude (β: -0.53, 95% CI: -0.86; -0.20 cm).

The more gradual score of adherences based on the EAT-Lancet index also resulted in associations of similar magnitude to those in the main analyses (Supplemental Table 8). Those with the greatest adherence to the EAT-Lancet index (23–42 points) had a 0.12 kg (95% CI: -0.29; 0.04 kg) lower follow-up weight compared to those with the lowest adherence to the EAT-Lancet index (0–17 points) when adjusting for baseline weight and confounders (model 1b). The estimated association was similar after adjusting for energy intake. In the WC-analyses, those with the greatest adherence to the EAT-Lancet index had a 0.42 cm (95% CI: -0.68; -0.16 cm) lower follow-up WC compared to those with the lowest EAT-Lancet index adherence when adjusting for confounders (model 1b). When further adjusting for energy intake, the association was of similar magnitude (β: -0.43, 95% CI: -0.69; -0.17, model 2).

The association between EAT-Lancet score and changes in weight without baseline adjustment showed no clear association (β: -0.01, 95% CI: -0.20; 0.19 kg, Table 3). When adjusting for baseline weight, the association showed a decrease in weight with highest compared to lowest EAT-Lancet score, albeit the CI included the null (β: -0.08, 95% CI: -0.27; 0.11 kg). The association between EAT-Lancet score and changes in WC without baseline adjustment showed a decrease in WC with highest compared to lowest EAT-Lancet score, albeit the CI was wide and included the null (β: -0.10, 95% CI: -0.41; 0.22, Table 3). When adjusting for baseline WC the association was significantly lower for those with highest compared to lowest EAT-Lancet adherence (β: -0.38, 95% CI: -0.69; -0.07 cm).

## 4 Discussion

The main results indicated no clear relation between EAT-Lancet score and body weight after five years. However, when assuming baseline weight a mediator of the association, a mean difference of 0.92 kg between the highest and lowest scoring participants was observed. In contrast, there was an inverse association, albeit weak, between higher compared to lower EAT-Lancet score and WC at follow-up in the main analyses. When considering baseline WC as mediator, the mean WC was 1.54 cm lower among the highest compared to lowest scoring participants. Adherence to the EAT-Lancet diet was associated with a lower risk of obesity and elevated WC after five years among those without obesity or elevated WC at baseline.

### 4.1 Strengths and limitations

This study has several strengths such as a large sample size and high data quality on many potential confounders of the association between EAT-Lancet score and development in weight and WC.

At the end of follow-up, 9121 (17.1%) eligible participants were excluded due to missing information on weight or WC, either because of incomplete questionnaires or because they did not return the questionnaire. A greater proportion of these had low, rather than high, EAT-Lancet scores. Loss to follow-up related only to the exposure would reduce the sample size and power. However, non-participation differential on the outcome cannot be ruled out [25], and could possibly explain the small magnitude of the results. Ibsen et al. [26] found that those who only completed the baseline assessment in this cohort and thus were lost to follow-up at the five-year data collection were more likely to be younger, have a lower education, consume less alcohol, be smokers, be less physically active, and have higher BMI and WC. Nevertheless, inverse probability of participation weighted analyses based on the characteristics of those who did not participate in the follow-up data collection in our study showed similar patterns of association as the main analyses. Excluding participants who developed diseases during follow-up resulted in associations of similar magnitude as in the main analyses, though with the caveat of introduced selection bias.

Although the questionnaire covering diet was validated [12,15], measurement error in the exposure is likely present. Misclassification of the exposure categories is most likely non-differential with regards to the outcome, biasing the estimate, on average, towards the null [25]. Adherence to the EAT-Lancet diet was based on a previous construct from the EPIC-Oxford study [14]. The diet score included two point-options; adherence or non-adherence to each of the 14 dietary components. This might categorize participants who are almost adherent with those who are far from adhering, hereby underestimating the association of interest. When scoring adherence on the more gradual EAT-Lancet index by Stubbendorff et al. [23], results were of similar magnitude as the main analyses indicating that the lack of association was not due to misclassification of the exposure in the crude EAT-Lancet diet scoring. Participants without an evident natural waist could be at risk of measuring WC differently at follow-up compared to baseline. Erroneous measurement of WC is most likely present across all exposure groups and therefore non-differential, which would on average bias estimates of association towards the null [25]. Results were robust to removal of outliers as the direction and magnitude of the associations did not change compared to the main analyses.

Despite adjusting extensively for potential confounders, residual confounding cannot be ruled out and might in part explain the small magnitude of the results. Other potential confounders have been proposed in other cohorts but were unmeasured in the DCH and could thus not be adjusted for in the analyses (Supplementary Fig. 1).

### 4.2 Adjustment for baseline measures and change analyses

Assuming reported diet at baseline a good representation of a stable and habitual diet before entry to the study is a strong assumption that relies on unobserved dietary habits as well as unobserved factors prior to study entry. Therefore, the primary analyses aimed to estimate the direct effect of the EAT-Lancet diet on follow-up weight and WC, conditioned on baseline weight and WC as well as potential confounders relevant to the follow-up period (Fig. 1, panel A). If participants’ diets were truly stable before entry, baseline weight and WC could be regarded as mediators of the association between EAT-Lancet adherence and follow-up weight and WC and should thus not be adjusted for (Fig. 1, panel B). Yet, without knowledge of participants’ diet prior to study entry, and thus the true temporal relation between habitual diet and baseline measures of weight and WC, the baseline measures could equally be mediators or confounders of the association between baseline diet and follow-up weight and WC. We therefore conducted analyses to test both assumptions. In the analyses without adjustment for baseline measures of the outcomes (Table 3), results showed a stronger association between EAT-Lancet diet and follow-up weight and WC as compared to the estimated direct effect with baseline adjustments. The results may cautiously be interpreted as an indication of general lower weight and WC for individuals with the highest adherence to the EAT-Lancet diet compared to those with the lowest.

A change-analysis adjusted for baseline is numerically equivalent to follow-up measure adjusted for baseline. Estimating change-measures without baseline adjustment may not have a causal interpretation as the causal pathways involved are mixed, because the outcome is in part predicted by the exposure before baseline [19]. Nevertheless, we also computed these models to improve the comparability of our findings with other research. The results (Table 3) showed no significant association between EAT-Lancet adherence and changes in weight or WC.

### 4.3 Adjustment for energy intake

The scoring of the EAT-Lancet diet (Supplementary Table 1) might introduce bias of the estimated association when adjusting for total energy intake, since some of the diet components do not have an upper limit or are scored based on a ratio of intakes. Adjusting for total energy intake (model 2) introduces a substitution aspect where individuals with different levels of EAT-Lancet adherence, but similar energy intakes, have consumed different amounts of the diet components with no upper limits or no specified amount, which may affect weight and WC development independently. However, the energy-adjusted analyses resulted in associations of similar magnitudes as the main analyses (Table 2).

### 4.4 Previous research

Smith et al. [27] evaluated three different methods for investigating diet in relation to long-term weight gain in prospective cohorts: baseline diet and weight change after four years, concurrent changes in diet and weight over four years, and changes in diet over four years with subsequent changes in weight over additional four years. Only the approach assessing concurrent changes in diet and weight resulted in significant associations. Nonetheless, as changes in diet and weight were measured simultaneously, there is also a risk of reverse causality [27], which was eliminated in our study by assessing diet at baseline and weight and WC after five years. However, the small magnitude of the results could in part be explained by unmeasured changes in dietary pattern happening concurrently with a change in weight and WC during follow-up.

The general tendency from observational research is that individuals adhering to plant-based dietary patterns have lower BMI, weight, and WC, and tend to gain less weight over time compared to omnivores [[28], [29], [30]]. We found that the relative risk of developing obesity at follow-up, defined as BMI ≥ 30 kg/m^2^ or WC above the WHO-defined cut-offs for men and women, was slightly lower for those with the highest compared to the lowest adherence to the EAT-Lancet diet. It is plausible that the small magnitude of associations in this current study would also be generalizable to other population where dietary patterns are stable.

### 4.5 Public health implications

The EAT-Lancet diet is recommended as healthy for both the planet and the global population. Yet, the diet remains to be tested regarding long-term development in weight and WC. The results of this study indicate that the diet can be beneficial for management of weight and WC in a population health perspective, particularly among those with a healthy weight and WC. A randomized controlled trial (RCT) investigating the New Nordic Diet, which included reduced meat intake, against the average Danish diet found a weight loss during intervention [31,32]. Since participants tend to resume old habits after end of intervention trials [31,32], evaluating acceptability of the diet could help understand how and if individuals will maintain adherence to a diet with a lower environmental impact. Further, cohorts emulating such interventions but with longer follow-up periods and multiple measures of diet and weight and WC over time could provide additional knowledge about the association between long-term adherence to the EAT-Lancet diet and weight and WC [33].

## 5 Conclusion

This study indicates that highest compared to lowest adherence to the EAT-Lancet diet was not associated with a difference in weight at follow-up but was associated with a slightly lower follow-up WC when adjusting for baseline weight and WC. Under the assumption that the association was mediated through baseline-measures of weight and WC, there were inverse associations between the EAT-Lancet diet and both weight and WC, with a considerably greater magnitude than the baseline-adjusted analyses. Greater adherence was also associated with lower risk of developing obesity or elevated WC. In sum, our findings suggested that greater adherence to the EAT-Lancet diet does not contribute to development of obesity.

## Author contributions

KO and AT were initiators and principal investigators of The Danish Diet, Cancer and Health cohort. CCD, DBI, FL and AO conceived the research question. CCD, FL and DBI designed the analysis plan. FL and DBI did the data analysis. FL, DBI and CCD wrote the paper. CCD had primary responsibility for the final content. All authors read and approved the final manuscript. interpreted the results and critically revised the article for important intellectual content and gave final approval of the version to publish.

## Funding

The 10.13039/100008363Danish Cancer Society funded the Diet, Cancer, and Health Cohort study. This study was funded by 10.13039/100007605Aarhus University. DBI was supported by a research grant from the 10.13039/501100004836Independent Research Fund Denmark (1057-00016B). The sponsors had no role in the design and conduct of the study; the collection, management, analysis, and interpretation of the data; or the preparation, review, or approval of the manuscript.

## Ethical approvals

The Danish Data Protection Agency and the local ethical committees of Copenhagen and Frederiksberg Municipalities (in Danish: "Den Videnskabsetiske Komite for Københavns og Frederiksberg Kommuner") approved the study with approval no.: (KF) 01-345/93. All participants gave written informed consent.

## Declaration of Competing Interest

The authors declare that they have no known competing financial interests or personal relationships that could have appeared to influence the work reported in this paper.

## Data Availability

Data described in this manuscript, code book, and analytical code will be made available upon reasonable request pending application and approval from the Danish Cancer Society (dchdata@cancer.dk). Questionnaires are available from the corresponding author.

## Appendix A Supplementary data

Supplementary material Image 8
