# Supplementary material for: Adherence to the EAT-Lancet diet in midlife and development in weight or waist circumference after five years in a Danish cohort
Source: Dialogues Health. 2023 Aug 19;3:100151. doi: 10.1016/j.dialog.2023.100151 (PMC10953849; doi:10.1016/j.dialog.2023.100151)
Supplement: Supplementary file 1 — Supplementary material [file mmc1.docx]

**Supplementary information: Adherence to the EAT-Lancet diet in midlife and development in weight or waist circumference after five years in a Danish cohort**

Fie Langmann^a^, Daniel B. Ibsen^a,b,c^, Anne Tjønneland^d,e^, Anja Olsen^a,c^, Kim Overvad^a^, Christina C. Dahm^a^

^a^Department of Public Health, Aarhus University, Bartholins Allé 2, 8000 Aarhus, Denmark.

^b^Steno Diabetes Center Aarhus, Aarhus University Hospital, Palle-Juul Jensens Blvd. 11, Entrance A, 8200 Aarhus, Denmark.

^c^Department of Nutrition, Exercise and Sports, University of Copenhagen, Nørre Allé 51, 2100 Copenhagen, Denmark.

^d^Danish Cancer Society, Strandboulevarden 49, 2100 Copenhagen, Denmark.

^e^Department of Public Health, University of Copenhagen, Øster Farimagsgade 5, 1353 Copenhagen, Denmark.

FL: [fie@ph.au.dk](mailto:fie@ph.au.dk); DBI: [dbi@ph.au.dk](mailto:dbi@ph.au.dk); AT: [annet@cancer.dk](mailto:annet@cancer.dk); AO: [anja@ph.au.dk](mailto:anja@ph.au.dk); KO: [ko@ph.au.dk](mailto:ko@ph.au.dk); CCD: [ccd@ph.au.dk](mailto:ccd@ph.au.dk).

Content

[Supplemental Table 1 – EAT-Lancet scoring criteria 3](#_Toc137473824)

[Supplemental Table 2 – Follow-up weight and WC without outliers 4](#_Toc137473825)

[Supplemental Table 3 – Stratified analyses of weight 5](#_Toc137473826)

[Supplemental Table 4 – Stratified analyses of waist circumference 6](#_Toc137473827)

[Supplemental Table 5 – Inverse probability weighted analysis 7](#_Toc137473828)

[Supplemental Table 6 – Exclusion of participants developing disease during follow-up 8](#_Toc137473829)

[Supplemental Table 7 – EAT-Lancet diet index 9](#_Toc137473830)

[Supplemental Figure 1 – Directed Acyclic Graph (DAG) 10](#_Toc137473831)

[Supplemental Figure 2 – Spline analyses 12](#_Toc137473832)

[Supplemental Figure 3 – Spline analyses 13](#_Toc137473833)

[Supplemental references 14](#_Toc137473834)

# Supplemental Table 1 – EAT-Lancet scoring criteria

Supplemental Table 1. EAT-Lancet diet scoring criteria based on components from the Danish Diet, Cancer, and Health cohort food frequency questionnaire (N=44,296).

| **Dietary component** | **Dietary components from the DCH FFQ** | **1-point scoring criteria** | **% (n) scoring 1 point** |
| --- | --- | --- | --- |
| 1. Grains | Whole grains, refined grains | ≤ 464 g/day  whole grain fibre > 5 g | 99.71 (44,168) |
| 2. Tubers and starchy vegetables | Potatoes, fatty potatoes | ≤ 100 g/day | 34.38 (15,230) |
| 3. Vegetables | Leafy, fruiting, stalk, or root vegetables, cabbages, mushrooms, onion/garlic | ≥ 200 g/day | 34.31 (15,198) |
| 4. Fruits | Citrus fruits, other fruits | ≥ 100 g/day | 66.71 (29,550) |
| 5. Dairy foods | Skimmed milk, semi-skimmed milk, whole milk, buttermilk, cheese, whole-fat fermented, low-fat fermented | ≤ 500 g/day | 71.34 (31,601) |
| 6. Beef, lamb, pork | Red meat, processed meat | ≤ 28 g/day | 1.95 (862) |
| 7. Chicken, other poultry | Poultry | ≤ 58 g/day | 94.89 (42,033) |
| 8. Eggs | Eggs | ≤ 25 g/day | 57.71 (25,565) |
| 9. Fish | Fresh lean, medium-fat and fatty fish, processed lean, medium-fat, and fatty fish | ≤ 100 g/day | 96.83 (42,890) |
| 10. Dry beans, lentils, peas | Legumes | ≤ 100 g/day^1^ | 100 (44,296) |
| 11. Soy foods | Soy | ≤ 50 g/day | 100 (44,296) |
| 12. Peanuts or tree nuts | Nuts | ≥ 25 g/day | 0.39 (171) |
| 13. Added fats | Saturated, monounsaturated, and polyunsaturated fatty acids | Ratio of 0.8 for unsaturated: saturated fat intake | 99.43 (44,045) |
| 14. Added sugars | Added sugar | ≤ 31 g/day | 51.74 (22,918) |

^1^Raw, dry weight.

DCH, Danish Diet, Cancer and Health cohort

FFQ, food frequency questionnaire.

# Supplemental Table 2 – Follow-up weight and WC without outliers

Supplemental Table 2. Linear association between EAT-Lancet adherence and follow-up weight and waist circumference (WC) after removal of outliers^1^ (N=43,498).

|  | **β (95% CI)** | | | | |
| --- | --- | --- | --- | --- | --- |
| **EAT-Lancet score** | **0-7** | **8** | **9** | **10** | **11-14** |
| Mean weight difference at follow-up (kg) | -0.08 (-0.21, 0.06) | -0.27 (-0.37, -0.18) | -0.25 (-0.33, -0.17) | -0.36 (-0.45, -0.27) | -0.32 (-0.44, -0.21) |
| Model 1a^2^ | Reference | -0.29 (-0.45, -0.13) | -0.35 (-0.50, -0.19) | -0.55 (-0.71, -0.39) | -0.61 (-0.78, -0.43) |
| Model 1b^3^ | Reference | -0.17 (-0.33, -0.01) | -0.11 (-0.27, 0.04) | -0.18 (-0.34, -0.02) | -0.14 (-0.31, 0.05) |
| Model 2^4^ | Reference | -0.18 (-0.34, -0.02) | -0.13 (-0.28, 0.03) | -0.20 (-0.37, -0.03) | -0.16 (-0.35, 0.03) |
| Mean WC difference at  follow-up (cm) | 4.06 (3.84, 4.28) | 4.54 (4.39, 4.69) | 5.01 (4.88, 5.15) | 5.46 (5.31, 5.62) | 5.80 (5.60, 5.99) |
| Model 1a^2^ | Reference | 0.08 (-0.18, 0.33) | 0.21 (-0.03, 0.46) | 0.21 (-0.04, 0.46) | 0.03 (-0.25, 0.31) |
| Model 1b^3^ | Reference | -0.01 (-0.26, 0.25) | 0.04 (-0.21, 0.28) | -0.05 (-0.30, 0.21) | -0.30 (-0.59, -0.02) |
| Model 2^4^ | Reference | -0.05 (-0.31, 0.20) | -0.04 (-0.29, 0.21) | -0.15 (-0.41, 0.12) | -0.42 (-0.72, -0.13) |

^1^Outliers defined as individuals with WC < 30 cm at follow-up, change in WC from baseline to follow-up of ≥50 cm, or changes in weight from baseline to follow-up of ≥40 kg, n removed = 798. ^2^Multi-variable linear regression analyses adjusted for baseline weight or baseline WC respectively. ^3^Further adjusted for sex (male, female), age at inclusion (years), physical activity (≥30 min/day, <30 min/day of moderate-to-vigorous physical activity), education (vocational, short 1-2 years, medium 3-4 years, high >4 years), smoking status (never, former, current <15 g tobacco/day, current 15–25 g tobacco/day, current >25 g tobacco/day), alcohol intake (g/day, restricted cubic splines with 4 knots), and previous history of hypertension (yes, no, don’t know), hypercholesterolemia (yes, no, don’t know), diabetes (yes, no, don’t know), stroke (yes, no), and acute myocardial infarct (yes, no) before baseline. ^4^Further adjusted for energy intake (kJ/day, continuous).

# Supplemental Table 3 – Stratified analyses of weight

Supplemental Table 3. Stratified analyses of the association between adherence to the EAT-Lancet diet and weight after five years.

|  | **β (95% CI)** | | | | |
| --- | --- | --- | --- | --- | --- |
| **EAT-Lancet score** | **0-7** | **8** | **9** | **10** | **11-14** |
| Baseline weight  <median^1^ (N=22,080)  ≥median (N=22,114) | Reference  Reference | -0.28 (-0.48, -0.07)  0.01 (-0.27, 0.29) | -0.17 (-0.36, 0.03)  0.03 (-0.24, 0.30) | -0.31 (-0.52, -0.11)  -0.11 (-0.39, 0.17) | -0.20 (-0.43, 0.03)  0.03 (-0.29, 0.34) |
| Baseline BMI  <25 kg/m^2^ (N=19,784)  ≥25 kg/m^2^ (N=24,103 | Reference  Reference | -0.33 (-0.54, -0.11)  0.04 (-0.23, 0.30) | -0.22 (-0.42, -0.01)  0.05 (-0.21, 0.30) | -0.46 (-0.67, -0.24)  0.02 (-0.25, 0.29) | -0.28 (-0.51, -0.04)  0.06 (-0.25, 0.37) |
| Age  <56 years (N=21,861)  ≥56 years (N=22,333) | Reference  Reference | -0.05 (-0.30, 0.20)  -0.13 (-0.38, 0.13) | 0.01 (-0.23, 0.26)  -0.10 (-0.34, 0.14) | -0.06 (-0.31, 0.20)  -0.32 (-0.58,- 0.07) | 0.05 (-0.23, 0.32)  -0.25 (-0.53, 0.04) |
| Sex  Male (N=20,789)  Female (N=23,405) | Reference  Reference | -0.16 (-0.45, 0.14)  -0.09 (-0.30, 0.13) | -0.05 (-0.33 0.23)  -0.05 (-0.26, 0.16) | -0.23 (-0.51, 0.05)  -0.15 (-0.39, 0.08) | -0.10 (-0.39, 0.19)  -0.08 (-0.38, 0.22) |

Multi-variable linear regression analyses adjusted for sex (male, female), age at inclusion (years), physical activity (≥30 min/day, <30 min/day of moderate-to-vigorous physical activity), education (vocational, short 1-2 years, medium 3-4 years, high >4 years), smoking status (never, former, current <15 g tobacco/day, current 15–25 g tobacco/day, current >25 g tobacco/day), alcohol intake (g/day, restricted cubic splines with 4 knots), and previous history of hypertension (yes, no, don’t know), hypercholesterolemia (yes, no, don’t know), diabetes (yes, no, don’t know), stroke (yes, no), and acute myocardial infarct (yes, no) before baseline. ^1^81.7 kg for men, 67 kg for women.

# Supplemental Table 4 – Stratified analyses of waist circumference

Supplemental Table 4. Stratified analyses of the association between adherence to the EAT-Lancet diet and waist circumference (WC) after five years.

|  | **β (95% CI)** | | | | |
| --- | --- | --- | --- | --- | --- |
| **EAT-Lancet score** | **0-7** | **8** | **9** | **10** | **11-14** |
| Baseline WC  <cut-offs^1^ (N=20,285)  ≥cut-offs (N=23,393) | Reference  Reference | -0.32 (-0.70, 0.05)  0.09 (-0.32, 0.50) | -0.23 (-0.59, 0.14)  0.00 (-0.40, 0.39) | -0.37 (-0.75, 0.01)  -0.01 (-0.43, 0.40) | -0.52 (-0.93, -0.11)  -0.24 (-0.71, 0.24) |
| Baseline BMI  <25 kg/m^2^ (N=19,575)  ≥25 kg/m^2^ (N=24,103) | Reference  Reference | -0.32 (-0.70, 0.05)  0.02 (-0.38, 0.42) | -0.23 (-0.59, 0.13)  -0.03 (-0.41, 0.36) | -0.42 (-0.79, -0.04)  -0.05 (-0.45, -0.36) | -0.57 (-0.97, -0.16)  -0.24 (-0.70, 0.23) |
| Age  <56 years (N=21,589)  ≥56 years (N=22,089) | Reference  Reference | -0.11 (-0.51, 0.29)  0.00 (-0.40, 0.41) | -0.13 (-0.52, 0.25)  -0.04 (-0.43, 0.35) | -0.19 (-0.59, 0.22)  -0.18 (-0.59, 0.23) | -0.41 (-0.85, 0.04)  -0.20 (-0.67, 0.27) |
| Sex  Male (N=20,597)  Female (N=23,081) | Reference  Reference | -0.04 (-0.55, 0.47)  -0.19 (-0.50, 0.12) | 0.11 (-0.37, 0.59)  -0.29 (-0.59, 0.01) | -0.13 (-0.61, 0.35)  -0.18 (-0.51, 0.16) | -0.29 (-0.79, 0.22)  -0.27 (-0.70, 0.16) |

Multi-variable linear regression analyses adjusted for sex (male, female), age at inclusion (years), physical activity (≥30 min/day, <30 min/day of moderate-to-vigorous physical activity), education (vocational, short 1-2 years, medium 3-4 years, high >4 years), smoking status (never, former, current <15 g tobacco/day, current 15–25 g tobacco/day, current >25 g tobacco/day), alcohol intake (g/day, restricted cubic splines with 4 knots), and previous history of hypertension (yes, no, don’t know), hypercholesterolemia (yes, no, don’t know), diabetes (yes, no, don’t know), stroke (yes, no), and acute myocardial infarct (yes, no) before baseline. ^1^Sex specific cut-offs for increased risk of cardiovascular disease specified by the World Health Organization, 94 cm for men, 80 cm for women.

# Supplemental Table 5 – Inverse probability weighted analysis

Supplemental Table 5. Association between EAT-Lancet score and weight and waist circumference (WC) at follow-up dependent on baseline weight and baseline WC in inverse probability weighted analyses.

|  | **β (95% CI)** | | | | |
| --- | --- | --- | --- | --- | --- |
| **EAT-Lancet score** | **0-7** | **8** | **9** | **10** | **11-14** |
| Mean weight difference at follow-up (kg)^1^ | -0.07 (-0.21, 0.06) | -0.22 (-0.32, -0.12) | -0.20 (-0.29, -0.11) | -0.38 (-0.47, -0.29) | -0.27 (-0.39, -0.15) |
| Model 1a^2^ | Reference | -0.27 (-0.44, -0.10) | -0.31 (-0.48, -0.14) | -0.59 (-0.77, -0.43) | -0.58 (-0.77, -0.39) |
| Model 1b^3^ | Reference | -0.13 (-0.30, 0.04) | -0.05 (-0.22, 0.11) | -0.20 (-0.37, 0.03) | -0.07 (-0.26, 0.12) |
| Model 2^4^ | Reference | -0.15 (-0.32, 0.03 | -0.07 (-0.25, 0.10) | -0.23 (-0.40, -0.05) | -0.10 (-0.30, 0.10) |
| Mean WC difference at follow-up (cm)^5^ | 4.29 (4.04, 4.54) | 4.65 (4.48, 4.81) | 5.10 (4.95, 5.24) | 5.53 (5.37, 5.69) | 5.94 (5.73, 6.15) |
| Model 1a^2^ | Reference | -0.06 (-0.36, 0.24) | 0.08 (-0.20, 0.37) | 0.08 (-0.22, 0.37) | -0.04 (-0.36, 0.29) |
| Model 1b^3^ | Reference | -0.14 (-0.43, 0.16) | -0.09 (-0.37, 0.20) | -0.18 (-0.47, 0.12) | -0.36 (-0.69, -0.03) |
| Model 2^4^ | Reference | -0.17 (-0.47, 0.12 | -0.14 (-0.43, 0.14) | -0.25 (-0.56, 0.05) | -0.45 (-0.79, -0.11) |

^1^N=44,192 included in weight analyses. ^2^Multi-variable linear regression analyses adjusted for baseline measures of weight or WC respectively. ^3^Further adjusted for sex (male, female), age at inclusion (years), physical activity (≥30 min/day, <30 min/day of moderate-to-vigorous physical activity), education (vocational, short 1-2 years, medium 3-4 years, high >4 years), smoking status (never, former, current <15 g tobacco/day, current 15–25 g tobacco/day, current >25 g tobacco/day), alcohol intake (g/day, restricted cubic splines with 4 knots), and previous history of hypertension (yes, no, don’t know), hypercholesterolemia (yes, no, don’t know), diabetes (yes, no, don’t know), stroke (yes, no), and acute myocardial infarct (yes, no) before baseline. ^4^Further adjusted for energy intake (kJ/day, continuous). ^5^N=43,675 included in WC analyses.

# Supplemental Table 6 – Exclusion of participants developing disease during follow-up

Supplemental Table 6. Association between EAT-Lancet score and weight and waist circumference (WC) at follow-up dependent on baseline weight and baseline WC after excluding participants who develop disease during follow-up^1^.

|  | **β (95% CI)** | | | | |
| --- | --- | --- | --- | --- | --- |
| **EAT-Lancet score** | **0-7** | **8** | **9** | **10** | **11-14** |
| Mean weight difference at follow-up (kg)^2^ | 0.07 (-0.07, 0.20) | -0.07 (-0.17, 0.03) | -0.10 (-0.19, 0.00) | -0.29 (-0.38, -0.20) | -0.21 (-0.33, -0.09) |
| Model 1a^3^ | Reference | -0.23 (-0.40, -0.05) | -0.31 (-0.48, -0.14) | -0.58 (-0.76, -0.41) | -0.59 (-0.78, -0.40) |
| Model 1b^4^ | Reference | -0.10 (-0.28, 0.07) | -0.08 (-0.24, 0.09) | -0.22 (-0.39, -0.04) | -0.12 (-0.31, 0.08) |
| Model 2^5^ | Reference | -0.11 (-0.28, 0.07) | -0.09 (-0.26, 0.09) | -0.23 (-0.42, -0.05) | -0.14 (-0.34, 0.07) |
| Mean WC difference at follow-up (cm)^6^ | 4.49 (4.23, 4.75) | 4.78 (4.61, 4.95) | 5.21 (5.07, 5.36) | 5.60 (5.44, 5.77) | 6.05 (5.84, 6.26) |
| Model 1a^3^ | Reference | -0.13 (-0.41, 0.16) | 0.00 (-0.27: 0.27) | -0.03 (-0.31, 0.26) | -0.10 (-0.41: 0.21) |
| Model 1b^4^ | Reference | -0.21 (-0.50, 0.07) | -0.17 (-0.44, 0.10) | -0.27 (-0.56, 0.01) | -0.42 (-0.73, -0.10) |
| Model 2^5^ | Reference | -0.26 (-0.54, 0.03) | -0.24 (-0.52, 0.04) | -0.37 (-0.66, -0.07) | -0.53 (-0.86, -0.20) |

^1^Exclusion of participants who developed diabetes, acute myocardial infarct, stroke, or colorectal cancer between baseline and follow-up, n excluded=2207. ^2^N=41,992 included in weight analyses. ^3^Multi-variable linear regression analyses adjusted for baseline weight or baseline WC, respectively. ^4^Further adjusted for sex (male, female), age at inclusion (years), physical activity (≥30 min/day, <30 min/day of moderate-to-vigorous physical activity), education (vocational, short 1-2 years, medium 3-4 years, high >4 years), smoking status (never, former, current <15 g tobacco/day, current 15–25 g tobacco/day, current >25 g tobacco/day), alcohol intake (g/day, restricted cubic splines with 4 knots), and previous history of hypertension (yes, no, don’t know), hypercholesterolemia (yes, no, don’t know), diabetes (yes, no, don’t know), stroke (yes, no), and acute myocardial infarct (yes, no) before baseline. ^5^Further adjusted for energy intake (kJ/day, continuous). ^6^N=41,517 included in WC analyses.

# Supplemental Table 7 – EAT-Lancet diet index

Supplemental Table 7. Association between EAT-Lancet diet index score and weight and waist circumference (WC) at follow-up dependent on baseline weight and baseline WC.

|  | **β (95% CI)** | | | | |
| --- | --- | --- | --- | --- | --- |
| **EAT-Lancet index score** | 0-17, n=7227 | 18-19, n=10,632 | 20-21, n=12,926 | 22, n=5188 | 23-42, n=8358 |
| Mean weight difference at follow-up (kg)^1^ | -0.07 (-0.21, 0.07) | -0.22 (-0.33, -0.12) | -0.20 (-0.29, -0.11) | -0.38 (-0.47, -0.29) | -0.27 (-0.39, -0.15) |
| Model 1a^2^ | Reference | -0.30 (-0.45, -0.15) | -0.33 (-0.48, -0.19) | -0.50 (-0.67, -0.32) | -0.55 (-0.71, -0.40) |
| Model 1b^3^ | Reference | -0.13 (-0.28, 0.02) | -0.06 (-0.21, 0.09) | -0.13 (-0.32, 0.05) | -0.12 (-0.29, 0.04) |
| Model 2^4^ | Reference | -0.13 (-0.28, 0.02) | -0.06 (-0.21, 0.08) | -0.14 (-0.32, 0.05) | -0.12 (-0.29, 0.04) |
| Mean WC difference at follow-up (cm)^5^ | 4.27 (4.02, 4.52) | 4.62 (4.46, 4.79) | 5.08 (4.94, 5.23) | 5.51 (5.35, 5.68) | 5.92 (5.71, 6.13) |
| Model 1a^2^ | Reference | -0.06 (-0.30, 0.18) | -0.24 (-0.47, -0.01) | -0.39 (-0.68, -0.11) | -0.42 (-0.67, -0.16) |
| Model 1b^3^ | Reference | -0.03 (-0.27, 0.20) | -0.18 (-0.42, 0.05) | -0.35 (-0.64, -0.07) | -0.42 (-0.68, -0.16) |
| Model 2^4^ | Reference | -0.04 (-0.27, 0.20) | -0.19 (-0.42, 0.05) | -0.36 (-0.65, -0.07) | -0.43 (-0.69, -0.17) |

^1^N=44,229 included in weight analyses. ^2^Multi-variable linear regression analyses adjusted for baseline weight or baseline WC, respectively. ^3^Further adjusted for sex (male, female), age at inclusion (years), physical activity (≥30 min/day, <30 min/day of moderate-to-vigorous physical activity), education (vocational, short 1-2 years, medium 3-4 years, high >4 years), smoking status (never, former, current <15 g tobacco/day, current 15–25 g tobacco/day, current >25 g tobacco/day), alcohol intake (g/day, restricted cubic splines with 4 knots), and previous history of hypertension (yes, no, don’t know), hypercholesterolemia (yes, no, don’t know), diabetes (yes, no, don’t know), stroke (yes, no), and acute myocardial infarct (yes, no) before baseline. ^4^Further adjusted for energy intake (kJ/day, continuous). ^5^N=43,710 included in WC analyses.

# Supplemental Figure 1 – Directed Acyclic Graph (DAG)


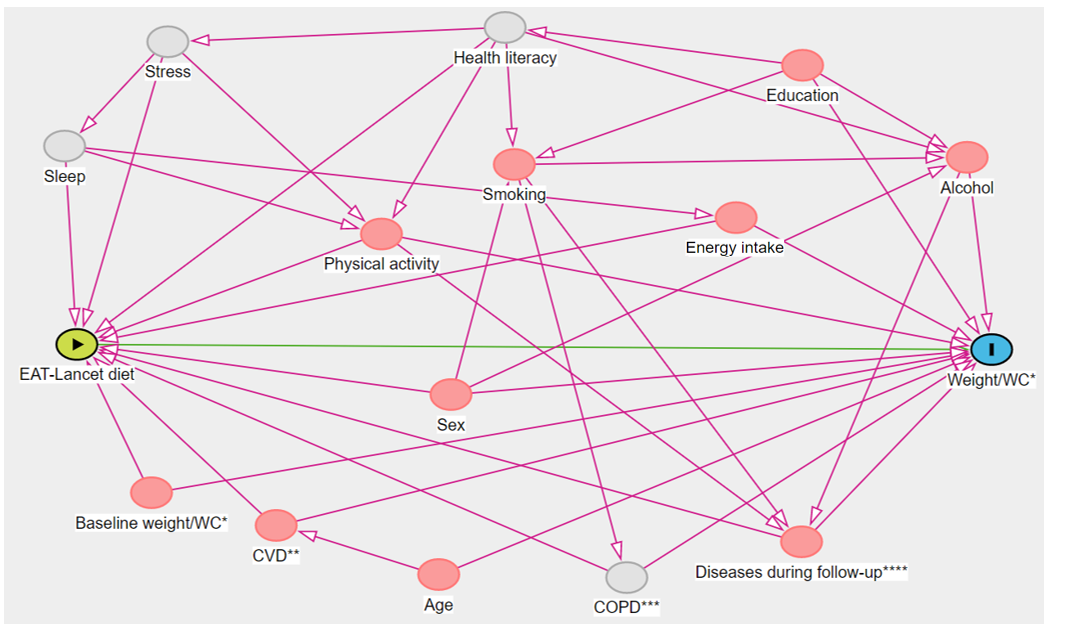


Supplemental Figure 1. Directed Acyclic Graph (DAG) of a priori presumed confounders of the association between EAT-Lancet diet and changes in weight and waist circumference. This DAG is constructed in Darity [1]. *WC: Waist circumference: **CVD: Previous history of cardiovascular diseases: hypertension, hypercholesterolemia, diabetes, apoplexy, and acute myocardial infarct, ***COPD, chronic obstructive pulmonary disease, ****Diseases during follow-up: colorectal cancer, diabetes, myocardial infarct, stroke,
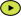
 exposure,
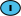
 outcome,
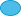
 ancestor of outcome,
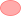
 ancestor of exposure and outcome,
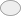
 unobserved (latent),
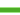
 causal path,
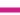
 biasing path.

**Adjusted DAG**

Based on the available data from the Danish Diet, Cancer and Health Cohort, the association between adherence to the EAT-Lancet diet and weight or WC was adjusted for physical activity, education, smoking, alcohol, age, sex, and CVD. Due to unknown dietary patterns of participants before entry to the study, we further adjusted for baseline weight and WC, as our data is limited to estimating the direct association between EAT-Lancet diet and follow-up weight and WC.

**Unmeasured confounders**

**Stress**

Stress affects individuals’ health behaviour, and a high stress level often results in poor health behaviours with increased risk of weight gain and low adherence to a healthy diet [2-5]. Stress can modify dietary intake and thereby risk of weight gain.

**Sleep**

Impaired sleep length or quality is associated with an increased appetite excessive of the actual energy need accounted by the extra awake hours [2, 6-8]. Poor sleep patterns are associated with higher intake of energy dense, processed foods with high amounts of sugar and fats [2, 4]. Some studies infer that acute sleep restriction decrease the individuals’ energy expenditure potentially leading to weight gain [9, 10]. Sleep patterns also have a direct effect on glucose metabolism and insulin resistance, and impaired sleep increases risk of onset of cardiovascular disease like type 2 diabetes [7, 10, 11].

**Health literacy**

Health literacy is affected by individuals’ sleep, where poor sleep patterns can result in lower health literacy and higher stress levels which may lead to poor health behaviours and weight gain as a consequence [12].

**Chronic obstructive pulmonary disease (COPD)**

Participants diagnosed with COPD might change their dietary habits and experience changes in body composition as a result of diagnosis and treatment. Thus, COPD may confound the association between diet and weight related outcomes [13, 14].

# Supplemental Figure 2 – Spline analyses


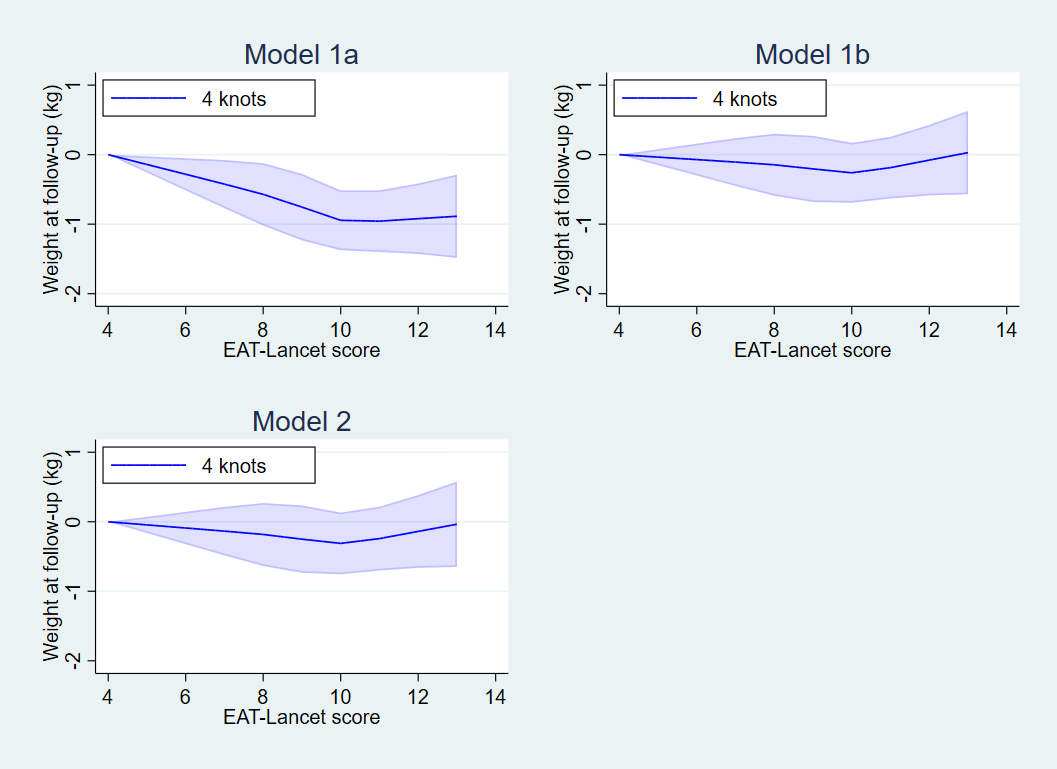


Supplemental Figure 2. Plot of EAT-Lancet score as restricted cubic splines with four knots against weight (kg) at follow-up. Model 1a: Adjusted for baseline weight. Model 1b: Further adjusted for sex (male, female), age (years), education (vocational, 1-2 years, 3-4 years, >4 years), physical activity ≥30 min/day (yes, no), smoking status (never, previous, current), alcohol intake (g/day), and previous history of hypertension (yes, no, don’t know), hypercholesterolemia (yes, no, don’t know), diabetes (yes, no, don’t know), stroke (yes, no), and acute myocardial infarct (yes, no) before baseline. Model 2: Further adjusted for energy intake (kJ/day, continuous). The 95% CI are presented as light blue markings.

# Supplemental Figure 3 – Spline analyses


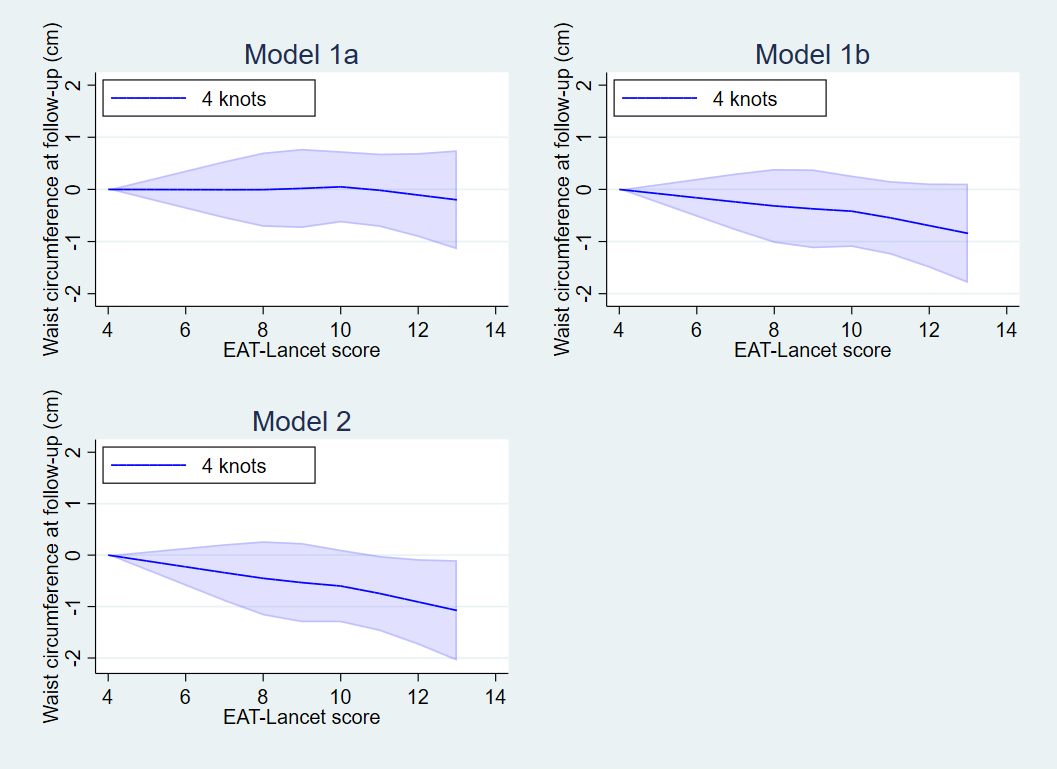


Supplemental figure 3. Plot of EAT-Lancet score as restricted cubic splines with four knots against waist circumference (cm) at follow-up. Model 1a: Adjusted for baseline WC. Model 1b: Further adjusted for sex (male, female), age (years), education (vocational, 1-2 years, 3-4 years, >4 years), physical activity ≥30 min/day (yes, no), smoking status (never, previous, current), alcohol intake (g/day), and previous history of hypertension (yes, no, don’t know), hypercholesterolemia (yes, no, don’t know), diabetes (yes, no, don’t know), stroke (yes, no), and acute myocardial infarct (yes, no) before baseline. Model 2: Further adjusted for energy intake (kJ/day, continuous). The 95% CI are presented as light blue markings.

# **Supplemental references**

1. Textor, J., van der Zander, B., Gilthorpe, M. K., Liskiewicz, M., Ellison, G. T. H., *Robust causal inference using directed acyclic graphs: the R package 'dagitty'.* International Journal of Epidemiology, 2016. **45**(6): p. 1887-1894.<https://doi.org/10.1093/ije/dyw341>

2. Spiegel, K., E. Tasali, P. PenevandE. Van Cauter, *Brief communication: Sleep curtailment in healthy young men is associated with decreased leptin levels, elevated ghrelin levels, and increased hunger and appetite.* Ann Intern Med, 2004. **141**(11): p. 846-50.<https://doi.org/10.7326/0003-4819-141-11-200412070-00008>

3. Jennum, P., Bonke, J., Clark, A. J., Flyvbjerg, A., Garde, A. H., Hermansen, K., Johansen, C., Møller, M., Rod, N. H., Sjödin, A., Zachariae, B., *Søvn og sundhed*. 2015, Vidensråd for forebyggelse: København. p. 1-224.

4. Chaput, J.P., J.P. Després, C. BouchardandA. Tremblay, *The association between short sleep duration and weight gain is dependent on disinhibited eating behavior in adults.* Sleep, 2011. **34**(10): p. 1291-7.<https://doi.org/10.5665/sleep.1264>

5. Block, J.P., Y. He, A.M. Zaslavsky, L. DingandJ.Z. Ayanian, *Psychosocial stress and change in weight among US adults.* Am J Epidemiol, 2009. **170**(2): p. 181-92.<https://doi.org/10.1093/aje/kwp104>

6. Schmid, S.M., M. Hallschmid, K. Jauch-Chara, J. BornandB. Schultes, *A single night of sleep deprivation increases ghrelin levels and feelings of hunger in normal-weight healthy men.* J Sleep Res, 2008. **17**(3): p. 331-4.<https://doi.org/10.1111/j.1365-2869.2008.00662.x>

7. Tasali, E., R. LeproultandK. Spiegel, *Reduced sleep duration or quality: relationships with insulin resistance and type 2 diabetes.* Prog Cardiovasc Dis, 2009. **51**(5): p. 381-91.<https://doi.org/10.1016/j.pcad.2008.10.002>

8. Van Cauter, E., K. Spiegel, E. TasaliandR. Leproult, *Metabolic consequences of sleep and sleep loss.* Sleep Med, 2008. **9 Suppl 1**(0 1): p. S23-8.<https://doi.org/10.1016/s1389-9457(08)70013-3>

9. Benedict, C., M. Hallschmid, A. Lassen, C. Mahnke, B. Schultes, H.B. Schiöth, et al., *Acute sleep deprivation reduces energy expenditure in healthy men.* The American Journal of Clinical Nutrition, 2011. **93**(6): p. 1229-1236.<https://doi.org/10.3945/ajcn.110.006460>

10. McNeil, J., É. DoucetandJ.P. Chaput, *Inadequate sleep as a contributor to obesity and type 2 diabetes.* Can J Diabetes, 2013. **37**(2): p. 103-8.<https://doi.org/10.1016/j.jcjd.2013.02.060>

11. Cappuccio, F.P., L. D'Elia, P. StrazzulloandM.A. Miller, *Quantity and quality of sleep and incidence of type 2 diabetes: a systematic review and meta-analysis.* Diabetes Care, 2010. **33**(2): p. 414-20.<https://doi.org/10.2337/dc09-1124>

12. Berkman, N.D., S.L. Sheridan, K.E. Donahue, D.J. HalpernandK. Crotty, *Low health literacy and health outcomes: an updated systematic review.* Ann Intern Med, 2011. **155**(2): p. 97-107.<https://doi.org/10.7326/0003-4819-155-2-201107190-00005>

13. Jakobsen, M.U., L. Madsen, C. Dethlefsen, K.M. Due, J. Halkjær, T.I.A. Sørensen, et al., *Dietary n-6 PUFA, carbohydrate:protein ratio and change in body weight and waist circumference: a follow-up study.* Public Health Nutrition, 2015. **18**(7): p. 1317-1323.<https://doi.org/10.1017/S1368980014001578>

14. Jakobsen, M.U., L. Madsen, F. Skjøth, T.L. Berentzen, J. Halkjær, A. Tjønneland, et al., *Dietary intake and adipose tissue content of long-chain n-3 PUFAs and subsequent 5-y change in body weight and waist circumference.* Am J Clin Nutr, 2017. **105**(5): p. 1148-1157.<https://doi.org/10.3945/ajcn.116.140079>
